# Effects of low frequency ultrasound on some properties of fibrinogen and its plasminolysis

**DOI:** 10.1186/1471-2091-12-60

**Published:** 2011-11-23

**Authors:** Eugene A Cherniavsky, Igor S Strakha, Igor E Adzerikho, Vladimir M Shkumatov

**Affiliations:** 1 Research Institute of Physical Chemical Problems, Belarusian State University, Leningradskaya Str., 220030, Minsk, Belarus; 2 Department of Clinical Pharmacology and Therapy, Belarusian Medical Academy of Postgraduate Education, 223040, Lesnoy, Minsk region, Belarus

## Abstract

**Background:**

Pharmacological thrombolysis with streptokinase, urokinase or tissue activator of plasminogen (t-PA), and mechanical interventions are frequently used in the treatment of both arterial and venous thrombotic diseases. It has been previously reported that application of ultrasound as an adjunct to thrombolytic therapy offers unique potential to improve effectiveness. However, little is known about effects of the ultrasound on proteins of blood coagulation and fibrinolysis. Here, we investigated the effects of the ultrasound on fibrinogen on processes of coagulation and fibrinogenolysis in an *in vitro *system.

**Results:**

Our study demonstrated that low frequency high intensity pulse ultrasound (25.1 kHz, 48.4 W/cm2, duty 50%) induced denaturation of plasminogen and t-PA and fibrinogen aggregates formation *in vitro*. The aggregates were characterized by the loss of clotting ability and a greater rate of plasminolysis than native fibrinogen. We investigated the effect of the ultrasound on individual proteins. In case of plasminogen and t-PA, ultrasound led to a decrease of the fibrinogenolysis rate, while it increased the fibrinogenolysis rate in case of fibrinogen. It has been shown that upon ultrasound treatment of mixture fibrinogen or fibrin with plasminogen, t-PA, or both, the rate of proteolytic digestion of fibrin(ogen) increases too. It has been shown that summary effect on the fibrin(ogen) proteolytic degradation under the conditions for combined ultrasound treatment is determined exclusively by effect on fibrin(ogen).

**Conclusions:**

The data presented here suggest that among proteins of fibrinolytic systems, the fibrinogen is one of the most sensitive proteins to the action of ultrasound. It has been shown *in vitro *that ultrasound induced fibrinogen aggregates formation, characterized by the loss of clotting ability and a greater rate of plasminolysis than native fibrinogen in different model systems and under different mode of ultrasound treatment. Under ultrasound treatment of plasminogen and/or t-PA in the presence of fibrin(ogen) the stabilizing effect fibrin(ogen) on given proteins was shown. On the other hand, an increase in the rate of fibrin(ogen) lysis was observed due to both the change in the substrate structure and promoting of the protein-protein complexes formation.

## Background

Thrombosis in the cardiovascular system is the one of the leading causes of mortality and morbidity in the world. Pharmacological thrombolysis using streptokinase, urokinase or tissue activator of plasminogen (t-PA) and mechanical interventions are frequently employed to treat both arterial and venous thrombotic diseases. Unfortunately, reperfusion is not always achieved and the success of the therapy is limited by reocclusion [1]. Limitations to these approaches also include complications such as bleeding, stroke, clot embolization and vessel wall damage [2]. Increasing evidence from *in vitro *[3-8], animal [9-11], and initial patients studies [12,13] indicates that application of ultrasound (US) as an adjunct to thrombolytic therapy offers unique potential to improve effectiveness and decrease bleeding complications. Now, four basic approaches to US thrombolysis have been pursued - two without pharmacological agents: (1) catheter-delivered external transducer US, (2) transcutaneous-delivered HIFU external US without drug delivery and US in conjunction with thrombolytic drugs and/or microbubbles or other agents, (3) catheter-delivered transducer-tipped US with local drug delivery, and (4) transcutaneous-delivered low frequency US with concomitant systemic (intravenous) drug delivery for site specific US augmentation [14]. Based on the available information the mechanisms involved in the fibrinolytic process in high-frequency, low-intensity US-enhanced fibrinolysis have been suggested [15] to be non drug-specific [16], not to alter the sizes of plasmatic derivatives or degradation products [17] and not to be caused by clot disruption [16]. US with a frequency of 1 MHz and intensities of 2.5-3.1 W/cm^2 ^had no statistically significant impact on biological activity of plasminogen activators: urokinase, streptokinase, alteplase and reteplase [18]. There is a limited data on the potential effect of US on proteins. It has been shown that high frequency (1 MHz) US with intensity of 6.92 W/cm^2 ^caused changes in structural order of cytochrome *c*, lysozyme, myoglobin, bovine serum albumin, trypsinogen and α-chymotrypsinogen A [19]. At low intensity US accelerates enzymatic thrombolysis through non-thermal mechanisms involving improvement in drug transport [8,20], reversibly altering fibrin structure [1,20] and increasing t-PA binding to fibrin [1]. Recent studies indicated that US at lower frequencies in the range of 20-40 kHz and impulse mode with duty cycles of 1 to 50% has a greater effect on thrombolysis with improved tissue penetration and less heating [1,11,21]. High intensity US disrupts clots into small fragments without administration of plasminogen activator [1,13]. The main mechanism by which ultrasonic lysis of thrombus occurs is termed the "cavitation effect." During the negative phase of the ultrasonic wave, microbubbles (or cavities) form within tissue exposed to the sound source. During the positive phase of the wave, these cavitations collapse, giving rise to a series of shock waves that lead to disruption and fragmentation. However, despite the documented efficiency, further clinical application of US angioplasty is restricted by the initiation of distal embolization of the resulting clot debris [14]. Our previously study demonstrated that at the conditions of a combined cavitation and streptokinase action at high US intensities, streptokinase preventing the formation of large clot debris conglomerates [22]. Based on this, we assume that the use of low frequency (20-40 kHz) impulse (duty cycle 1-50%) mild intensity (10-50 W/cm^2^) US in combination with thrombolytic drugs will make it possible to decrease the risk of side effects development. The effectiveness of this approach has been confirmed by our in animal study [23].

With a number of described above mechanisms of US thrombus destruction and US enhanced fibrinolysis additional mechanisms may underlie the effects of US thrombolysis Analyses of the products of the plasma clot destruction by the low-frequency ultrasound (27 kHz) showed that at the US intensities below 21.6 W/cm^2^, there was extraction of blood serum proteins located in the pores of the fibrin network. The increase in intensity of the ultrasonic treatment was accompanied by protofibrils disaggregation and extraction of blood serum proteins located inside fibrin fibers. Proteins extracted from the plasma clot underwent subsequent aggregation, whereas interaction between protofibrils caused formation of insoluble fibrin particles [24]. Thus, low frequency and high intensity ultrasound together with the macroscopic disruption of the clot can affect structural-functional properties of proteins.

It has been previously shown that usage of low frequency US (26 kHz) with intensity up to 15 W/cm^2 ^does not influence properties of streptokinase and antithrombin III. At US intensity exceeding 26 W/cm^2^, plasminogen activator streptokinase undergoes irreversible aggregation with unfolding of protein molecule and exposure of new peptide bonds susceptible for proteolytic attack by plasmin [25]. After US treatment, antithrombin III is converted into inactive forms (latent and polymeric aggregates), whereas non-activated blood clotting factors belonging to the α-chymotrypsin superfamily do not change their structural and functional parameters within PPSB complex [26]. At the same time, a functional model of activation of equimolar mixture of α-chymotrypsinogen and trypsinogen revealed that trypsin is the most sensitive to the US treatment [27].

The aim of current studies was to investigate the effects of low frequency high intensity pulse US (25.1 kHz, 48.4 W/cm2, duty 50%) on the key protein of the blood coagulation - fibrinogen, as well as to investigate the effects of US on the fibrinolysis in an *in vitro system*. It has been shown that ultrasound induced time-dependent formation of fibrinogen aggregates, characterized by loss of clotting ability and greater rate of plasminolysis than native fibrinogen and denaturation of plasminogen and t-PA. Under ultrasound treatment of plasminogen and/or t-PA in the presence of fibrin(ogen), the stabilizing effect of fibrin(ogen) on given proteins was demonstrated. On the other hand, an increase in the rate of fibrin(ogen) lysis was observed due to both the change in the substrate structure and promoting of the protein-protein complexes formation. Studies in this direction are address the key question how to minimize side effects and increase the effectiveness of ultrasonic trombolysis.

## Methods

### Materials

The following reagents were used in this study: SDS, 2-mercaptoethanol, Coomassie Brilliant Blue G-250, tris-(hydroxymethyl)aminomethane, N, N, N', N'- tetramethylenediamine, glycine, ammonium persulfate ("Serva", Germany);the mixture of the inhibitors of proteases ("Roche", Germany), N.N'-methylene-bis-acrylamide, ("Reanal", Hungary); the collection of protein standards for the gel-electrophoresis (phosphorylase B (97.4 kDa), bovine serum albumin (67 kDa), ovalbumin (45 kDa), carboanhydrase (29 kDa)) ("Serva", Germany); Lysine-Sepharose were obtained by the immobilization of lysine to epichlorohydrin-activated Sepharose-4B CL [28].

The t-PA was obtained from the manufacturer (recombinant t-PA, Actilyse, "Boehringer Ingelheim", Germany) was dissolved in 0.05 M Tris-HCl, 0.15 M NaCl (pH 7.4) to give a final concentration of 10 mg/ml and stored at -20°C. This solution was used for the activation of plasminogen preparations.

Bovine fibrinogen, 97% clottable, was prepared the method of Blomback and Blomback [29] from fresh frozen citrate plasma. Plasminogen was purified by affinity chromatography on lysine-Sepharose from fresh frozen citrated bovine plasma according to Deutsch et al. [30].

### Ultrasound source

The thrombolytic device "Pulsar" developed at the Belarusian State Polytechnic Academy (Minsk, Belarus) was used as a source of US. It consists of an US generator, a piezoelectric converter of the US oscillations, and a flexible 0.8 mm ball-tipped wire. The diameter of the emitting working surface is 0.6 mm with a length of 245 mm. The power output of generator is 80 W. The power output at the acoustic horn was 48.4 W/cm^2 ^with a 50% duty cycle of 0.9 sec on and 0.9 sec off and a frequency of 25.1 kHz.

The protein was dissolved in 0.05 M Tris-HCl buffer at pH 7.4, containing 0.15 M NaCl. The sample solution in a polypropylene tube was placed in a water bath at 37°C. The US wire was loaded in sample solution at a depth of 1 cm. During US treatment, temperature increase did not exceed 0.5°C.

### Gel-permeation chromatography

Chromatography was carried out on an automatic GradiFrac (Pharmacia Biotech) system including a programmed fraction collector, a flow UV-detector (λ = 280 nm), a gradient mixer, and a peristaltic pump. A Sepharose 4B-CL column (10 × 950 mm) was used. The buffer containing 0.05 M Tris-HCl at pH 7.4 and 0.15 M sodium chloride was used to equilibrate the column. Different fibrinogen species were eluted at a flow rate 0.1 ml/min.

### SDS-PAGE

Electrophoresis in 10% and 7.5% polyacrylamide with and without reduction with 5% (v/v) 2-mercaptoethanol was performed as described by Laemmli [31]. Protein bands were visualized with Coomassie Blue staining. The calculation of the molecular weights of bands and densitograms were constructed with using "Phoretix 1D" software (Nonlinear Dynamics Ltd, United States)

### Scanning electron microscopy

Clots obtained after the US treatment were fixed, dehydrated, dried and covered with an electrically conducting 15- 20 nm thick golden layer [22]. The photomicrograph was obtained by means of a Nanolab-7 scanning electron microscope using an accelerating voltage of 20 kV at magnifications of 1000×, 2500×, 5000× and 10000×.

### Turbidity measurements

Polymerization at the ambient temperature was monitored continuously at 350 nm in a spectrophotometer UV-1202 (Shimadzu Corporation, Tokyo, Japan). The reaction was initiated by addition of 300 μl thrombin at a concentration from 25 unit/ml to 3 ml fibrinogen solution (3 mg/ml) in 0.05 M Tris-HCl, pH 7.4, containing 0.15 M sodium chloride.

### Fibrinogenolysis

Fibrinogenolysis was carried out in 0.05 M Na-phosphate buffer, containing 0.15 M NaCl at pH 7.4. Proteolysis was initiated by addition of 150 μl of plasminogen (2 mg/ml) and t-PA (0.2 mg/ml) to 15 ml of the solution of fibrinogen (3 mg/ml). The digestion mixture was then incubated at 37°C and samples were collected after certain time intervals for SDS-PAGE and determination of TCA-soluble peptide analysis.

### Fibrinolysis

#### Clot preparation

The fibrin clot was prepared by an addition of 300 μl of 1 M CaCl_2 _and 1.5 ml of thrombin solution (25 unit/ml in 0.05 M Tris-HCl, 0.5 M NaCl at pH 7.4) to 15 ml fibrinogen solution (3 mg/ml in the same buffer). The final mixture was incubated for 2 hour at 37°C. The resultant clot was dried between the two sheets of the filter paper.

#### Clot lysis

The clot was placed in the polypropylene tube with buffer, containing 15 ml 0.05 M Tris-HCl and 0.5 M NaCl at pH 7.4. For the initiation of the fibrinolysis 150 μl of the plasminogen solution (1.5 mg/ml in 0,025 M Tris-HCl, 50% glycerol, pH 7,4) and 150 μl rt-PA (10 mg/ml in 0.05 M Tris-HCl, 0.5 M NaCl (pH 7.4)) were added to the mixture. The mixture was incubated at 37°C and during 4 hours of hydrolysis the samples were collected. The aliquots were used for SDS-PAGE (a mixture for protein denaturation with or without 2-mercaptoethanol was directly added to these aliquots) and the determination of TCA-soluble peptide.

### Determination of TCA-soluble peptide

To 1.5 ml analyzable sample 1.0 ml 15% TCA were added. Samples centrifuged (8000 × g, 5 min) and the optical density of solution at 280 nm was measured. The concentration of the TCA-dissoluble peptide they recounted to the appropriate concentration of tyrosine into μmole/L according to the formula:

$$\left[\text{Tyr}\right]={\text{D}}_{\text{28}0\;\text{nm}}\cdot \text{2}.\text{5}∕\text{1}.\text{15}$$

### Statistical analysis

Statistical analysis was carried out employed the Microsoft Excel 2000 software. Results were tested by the Student's *t *test. Data expressed as mean +/- standard deviation. A p value ≤ 0.05 was considered to be statistically significant.

## Results

**Figure 1:**
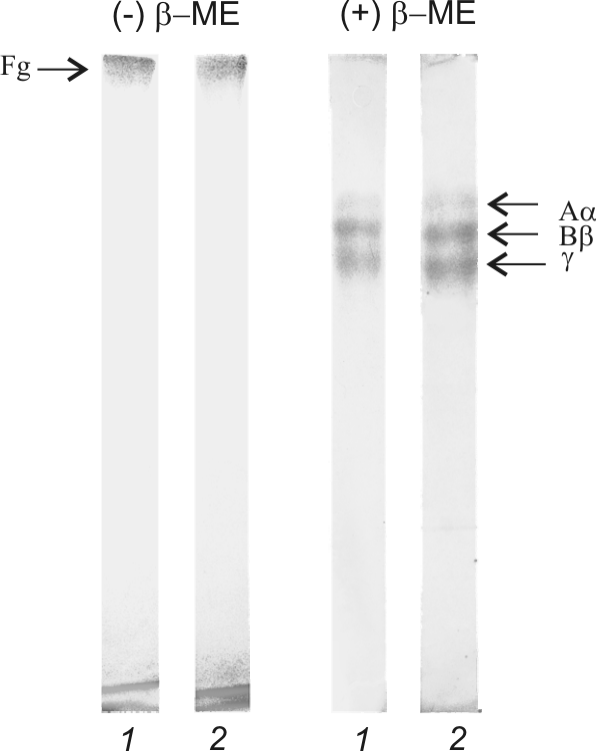
**SDS-PAGE analysis of fibrinogen samples**. *1 *- sonicated (25.1 kHz, 48.4 W/cm2, duty 50%) at 37°C for 30 min; *2 *- control (treated at 37°C for 30 min) samples of fibrinogen (Fg) in the presence (+βME) and absence (-βME) β-mercaptoethanol. Aα, Bβ, γ - corresponding native fibrinogen chains.

Fibrinogen was exposed to ultrasound for 30 minutes at 37°C and then assayed by SDS-PAGE under reducing and nonreducing conditions (Figure 1). Gel-electrophoresis without reduction of inter- and intramolecular disulfide bonds showed presence of one polypeptide with the molecular weight of more than 250 kDa (band on the top of the gel) in the US-treated and the control samples. The same samples separated on the SDS-PAGE gel containing β-mercaptoethanol showed three identical polypeptides corresponding to native Aα-, Bβ-, and γ-chains of fibrinogen (Figure 1).

**Figure 2:**
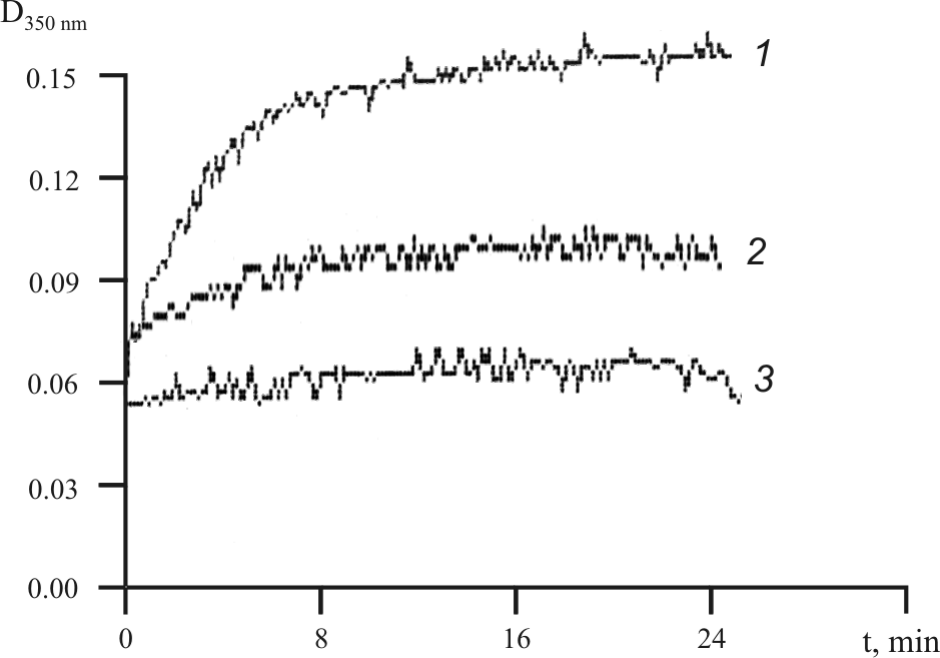
**Clotting of fibrinogen**. native (*1*) and US-treated (25.1 kHz, 48.4 W/cm2, duty 50%) during 5 (*2*) and 30 (*3*) min fibrinogen.

The effects of the US on functional activity of the fibrinogen were investigated. For this purpose, two physiologically important processes involving fibrinogen were tested: the formation of a fibrinogen clot and fibrinogenolysis. The clotting ability was tested by adding thrombin and measuring the increase in turbidity at 350 nm. For the control sample of fibrinogen, exponential increase of the turbidity with an increase of incubation time was observed (Figure 2, line *1*). The initial velocity and the stationary level of the absorption decreased for the sample treatment ultrasound during 5 min (Figure 2, line *2*). A sonication of 30 minutes led to the total loss of the clotting ability (Figure 2, line *3*).

**Figure 3:**
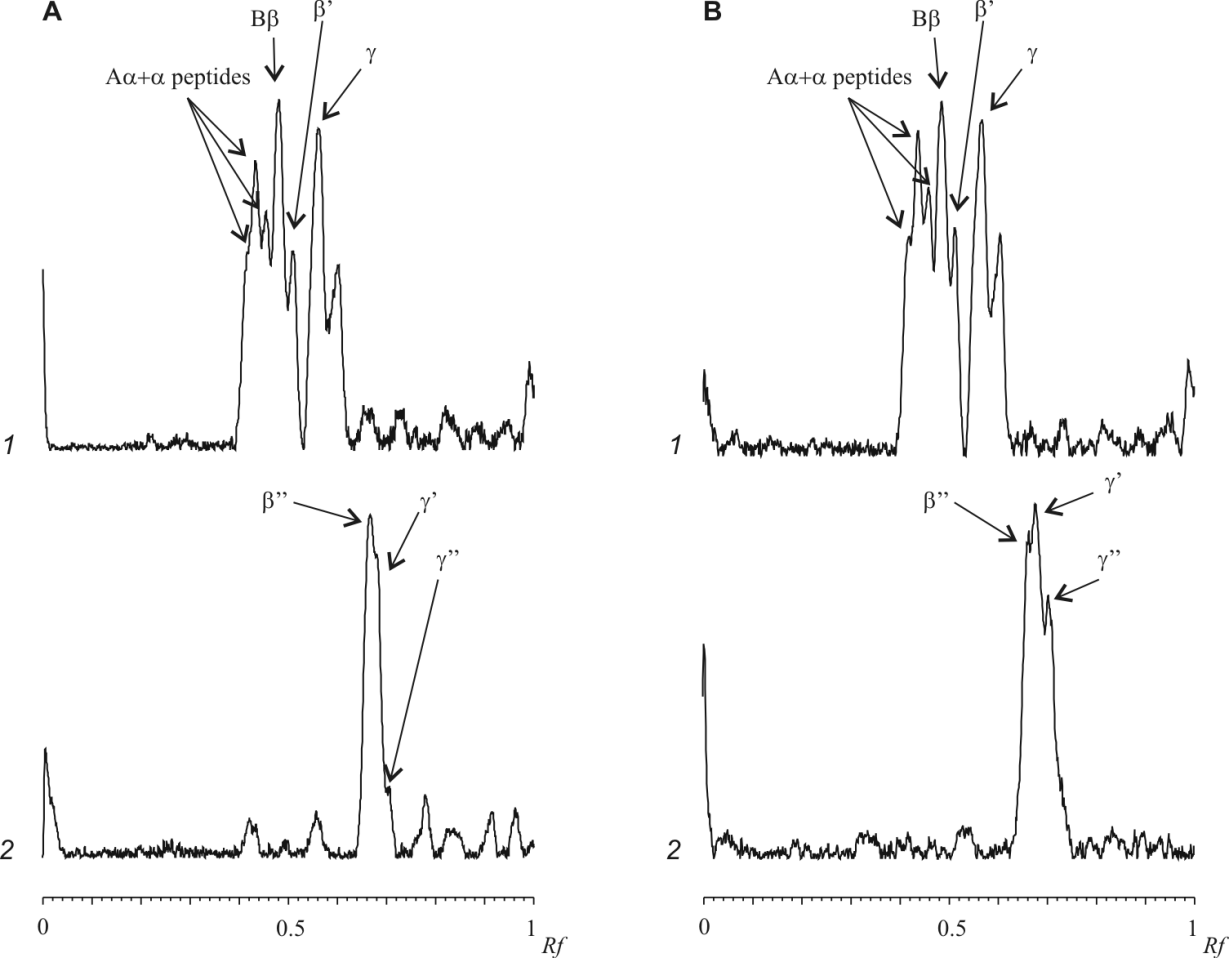
**Densitograms of SDS-PAGE analysis of fibrinogen proteolysis products in the presence of β-mercaptoethanol**. **A **- native and **B **- sonicated (25.1 kHz, 48.4 W/cm2, duty 50%,) at 37°C during 15 min fibrinogen, after 15 min (*1*) and 5 hours (*2*) after addition mixture of plasminogen with rt-PA. Aα, Bβ, β', β'', γ, γ', γ'' - corresponding chains of fibrinogen.

The electrophoretic pattern of the fibrinogen proteolytic degradation products by plasmin after treatment with β-mercaptoethanol is shown in Figure 3. Identical distribution of polypeptide bands for both sonicated and control fibrinogen samples were observed. At zero time of proteolysis, three polypeptides corresponding to Aα-, Bβ-, and γ-chains of fibrinogen were present. The decrease of the content of the native chains and respectively the accumulation of proteolytically modified β''-, γ'-, γ''- and γ'''- chains of fibrinogen were observed during the time course of the fibrinogen incubation with plasminogen and t-PA (Figure 3).

**Figure 4:**
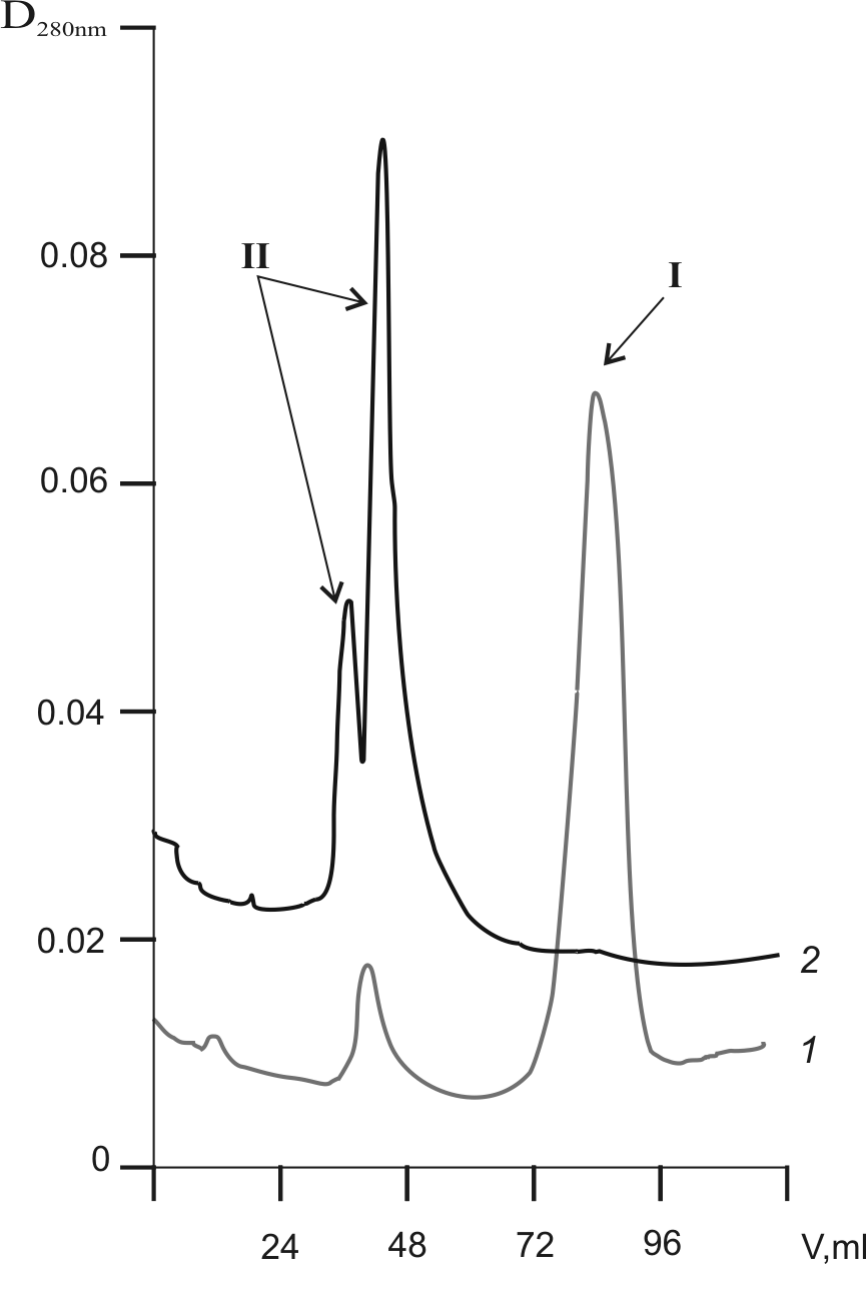
**Gel-permeation chromatography of fibrinogen samples**. *1 *- native; *2 *- US-treatment (25.1 kHz, 48.4 W/cm2, duty 50%,) during 30 min. Column: Sepharose-4B CL (950 × 16 mm), buffer 0,05 M Na-phosphate, 0,15 M NaCl, pH 7,4, rate 0,1 ml/min, detection 280 nm. I-fibrinogen, II-fibrinogen aggregates.

Figure 4 shows the results of fibrinogen gel-permeation chromatography before and after sonication. There are two peaks on the chromatogram of native fibrinogen. First dominant peak is native fibrinogen (MW 340 kDa), while second one corresponds to proteins with MW > 600 kDa (Figure 4, line *1*). In turn, on the chromatogram with the US-treated fibrinogen sample, there are two peaks corresponding to proteins with MW > 600 kDa (Figure 4, line *2*). The increase in the molecular weight of proteins occurs due to the formation of aggregates, since the US does not induce the formation of covalent cross-links as demonstrated above.

**Figure 5:**
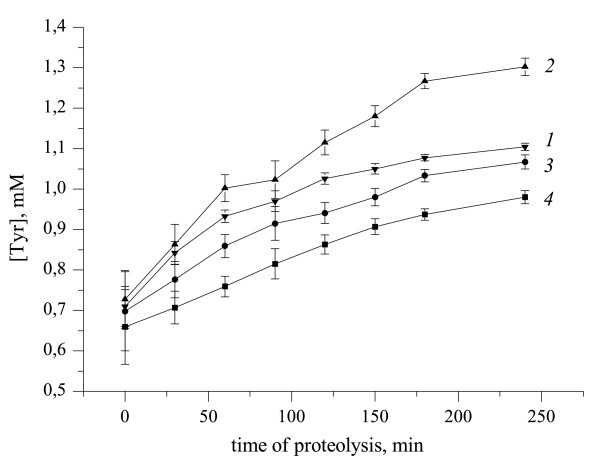
**Proteolytic degradation of fibrinogen by the mixture of plasminogen and rt-PA under different modes of US-treatment**. *1 *- control (without US action); *2 *- sonicated during 30 min fibrinogen; *3 *- sonicated during 30 min plasminogen; *4 *- sonicated during 30 min rt-PA. Ultrasound: 25.1 kHz, 48.4 W/cm2, duty 50%. Data represent mean +/-SD of three independent experiments.

Next, the influence of the low-frequency US on the proteolytic degradation of fibrinogen in the fibrinogen: plasminogen: t-PA system was investigated. The fibrinogenolysis rate was estimated by determining the concentration of TCA-soluble peptide released during proteins incubation at 37°C. Figure 5 shows that US has differential effects on individual proteins. The US treatment led to increase of fibrinogenolysis rate in case of fibrinogen (Figure 5, line *2*), while it decreased fibrinogenolysis in case of plasminogen (Figure 5, line *3*) and t- PA (Figure 5, line *4*) in comparison to control (Figure 5, line *1*).

**Figure 6:**
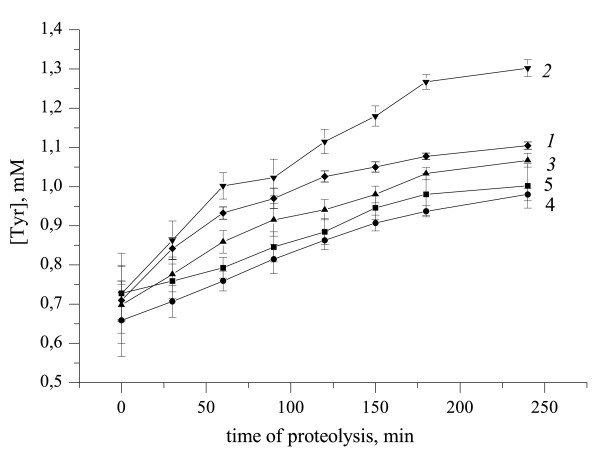
**Proteolytic degradation of fibrinogen by the mixture of plasminogen and rt-PA under different modes of US-treatment**. *1*-control; *2*- prior incubation rt-PA with plasminogen at 37°C during 30 min; *3*- sonicated during 30 min plasminogen; *4*- sonicated during 30 min t-PA; *5 *-prior US-treatment mixture of plasminogen with t-PA. Ultrasound: 25.1 kHz, 48.4 W/cm2, duty 50%. Data represent mean +/-SD of three independent experiments.

Figure 6 shows that the beforehand incubation of plasminogen with t- PA (Figure 6, line *2*) for 30 min led to increase in the fibrinogenolysis rate in comparison with the control experiment (Figure 6, line *1*). Obviously, in this case a larger quantity of already formed active plasmin was added to fibrinogen. In turn, beforehand US treatment of the mixture of plasminogen with t-PA leads to considerable reduction in the fibrinogenolysis rate (Figure 6, line *5*). Moreover, this effect was not the sum of the inactivating actions of ultrasound on t-PA (Figure 6, line *3*) and plasminogen (Figure 6, line *4*), but was taken intermediate value.

**Figure 7:**
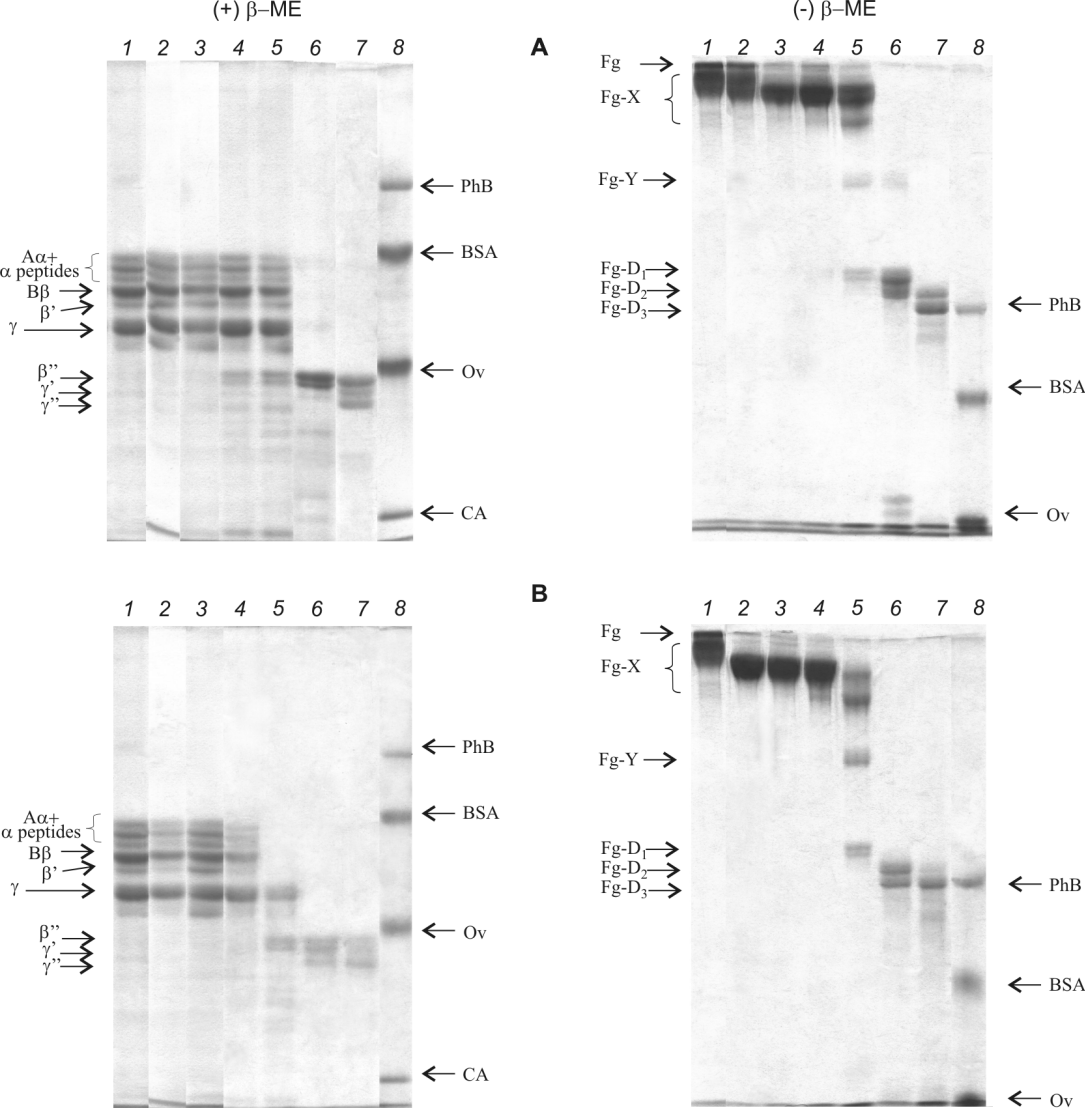
**Gel- electrophoresis of the fibrinogen degradation products in the model systems**. **A **- fibrinogen:plasminogen:t-PA, **B **- preliminary US treatment (25.1 kHz, 48.4 W/cm2, duty 50%) mixture of fibrinogen with plasminogen during 15 min. with the subsequent addition of rt-PA. *1,2,3,4,5,6,7 *- 0,5,15,30,60 min, 5 h, 24 h of hydrolysis, respectively; 8 - standards. β- ME - β - mercaptoethanol. Fg, Fg-X, Fg-Y, Fg-D_1_, Fg-D_2_, Fg-D_3 _are fibrinogen and products of its proteolytic degradation, respectively. Aα, Bβ, β', β'', γ, γ', γ'' - corresponding chains of fibrinogen. PhB - phosphorylase B, BSA - bovine serum albumin, Ov - ovalbumin, CA - carboanhydrase.

The influence of ultrasound on the rate of the enzymatic hydrolysis of fibrinogen in the fibrinogen: plasminogen: t-PA system was investigated upon introduction of the plasminogen activator after the US treatment. Figure 7 presents data analysis of reducing and non-reducing samples of fibrinogen proteolysis products. Time-dependent degradation of the native fibrinogen polypeptide chain (Aα, Bβ and γ) and accordingly the accumulation of the end products of the fibrinogen plasminolysis - fragments D_1_, D_2_, D_3 _through the intermediate formation of the partially degraded fragments X and Y occurred independently of the US treatment. However, the electrophoregrams of the not reducing control sample after 1 hour of the hydrolysis (Figure 7A, right column, line *5*) show the presence of only early fibrinogen degradation product - fragment X. At the same time, in the case of preliminary sonication of fibrinogen with plasminogen approximately half of all fibrinogen is converted into the fragments Y and D (Figure 7B, right column, line *5*).

**Figure 8:**
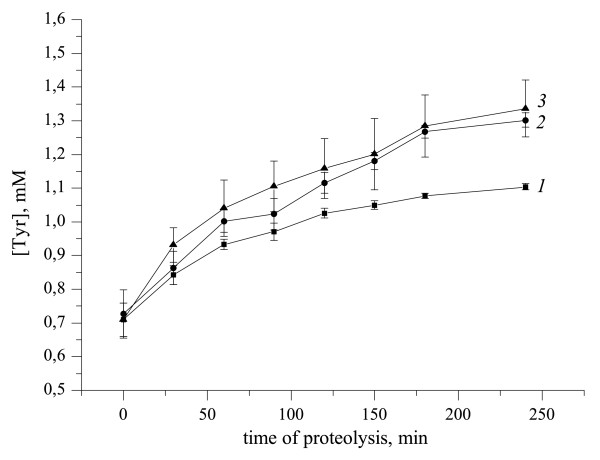
**Proteolytic degradation of fibrinogen by the mixture of plasminogen and rt-PA under different modes of US-treatment**. US - 25.1 kHz, 48.4 W/cm^2^, duty 50%. *1*-control; *2*- sonicated during 30 min fibrinogen; *3*- US-treatment mixture of fibrinogen, plasminogen and rt-PA. Data represent mean +/-SD of three independent experiments.

The following part concerns the investigation of the influence of ultrasound on fibrinogenolysis with the US treatment of all three proteins (Figure 8). It is evident that in the case of combined US-treatment (Figure 8, line *3*), an increase of fibrinogenolysis rate occurs as in case of fibrinogen.

**Figure 9:**
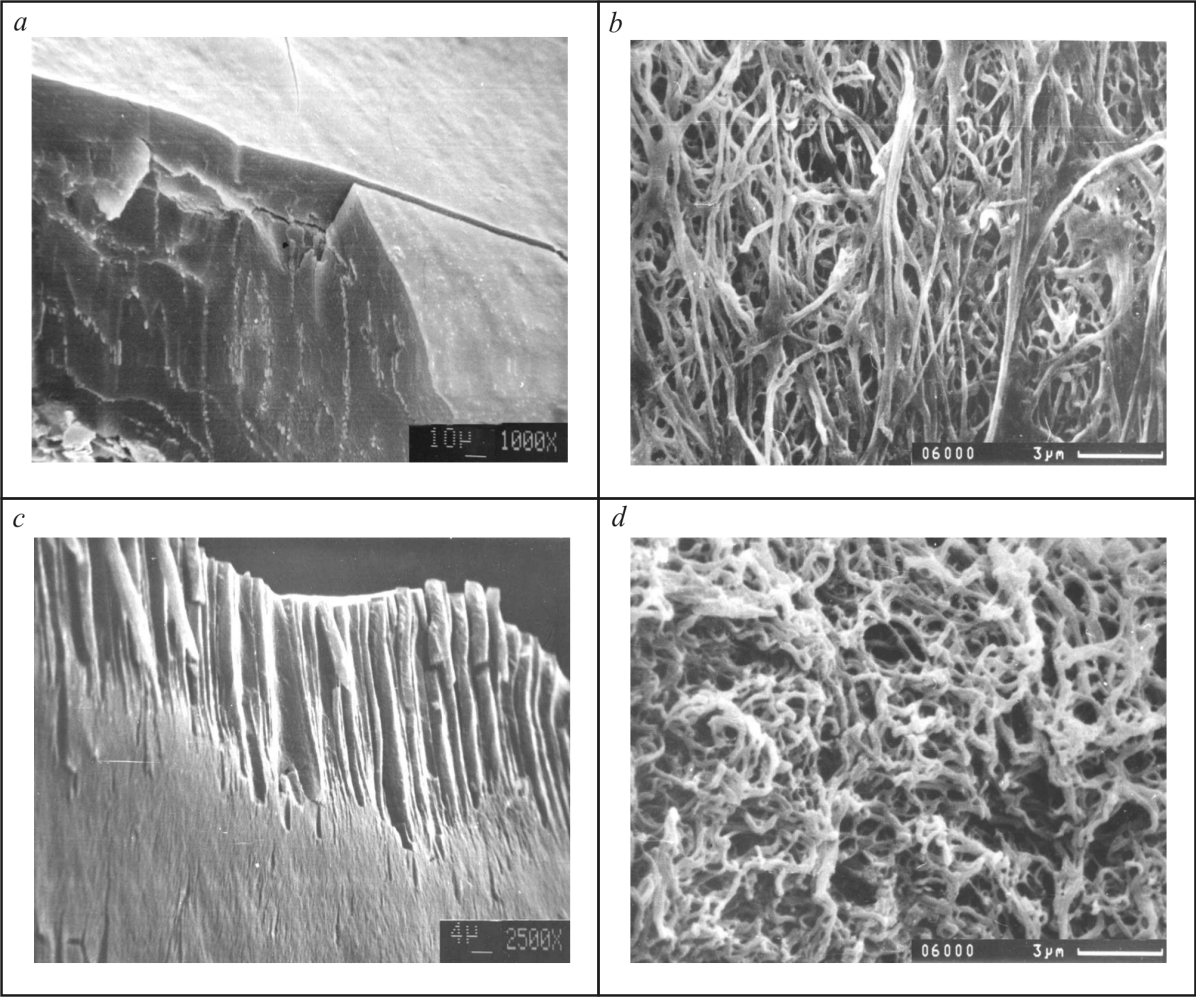
**Images of control (*a*, *b*) and US-treated (*c*, *d*) fibrin clot obtained by scanning electron microscopy at different magnifications**.

For the investigation of US effects on fibrinolysis, the model of fibrin clot, prepared by coagulation of pure fibrinogen by thrombin in presence of Ca^2+ ^ions, was used. Initial fibrinogen contained sufficient quantity of factor XIII for complete covalent cross-linking of fibrin monomers. Plasminolysis was initiated by addition of the plasminogen and t-PA to the fibrinogen solution. The process of the fibrinolysis, like fibrinogenolysis, was controlled by the determination of the concentration of TCA-soluble peptide formation. As in the case of fibrinogen, US-treatment leads to the increased rate of proteolysis in both cases, after treating the clot itself and clot in the presence of plasminogen and/or rt-PA. SDS-PAGE of fibrin degradation products revealed that already after 2 hour of hydrolysis mainly D-dimers were present. At the same time, in the degradation products of previously US-treated fibrin parallel with D-dimers early fibrin degradation products - fragments X and Y were present (data not shown). Analysis by the scanning electron microscopy (Figure 9) showed that clot revealed many deepening of 4 - 10 μm in size as a result of the US action on the surface of fibrin. The analysis of the internal structure of the clot showed that multiple channels appeared near the clot surface. These channels were 1-2 μm in diameter, and 20-45 μm in length (Figure 9. *c*). Changes were also observed in the fibrin network that acquired fine-mesh shape due to mach irregular disruptions of fibers (Figure 9. *d*). The quantitative analysis of the image showed substantial changes in parameters of the US-treated fibrin network compared to control. The ultrasound caused decrease of length and diameter of fibrin fibers, but at the same time increase in pore size.

## Discussion

This study demonstrated that the US did not cause the cleavage of peptide and interchain disulfide bonds or formation of interchain and intermolecular cross-links in case of fibrinogen (Figure 1). Additionally, results presented here suggested that the US did not induce the conformational changes in the fibrinogen molecule leading to the exposure of a new plasmin-sensitive site (Figure 3). Our results are consistent with previously reported data describing the effect of high-frequency US on fibrinogen [16] and fibrin clot [17]. However, sonication of fibrinogen with the US (25.1 kHz, 48.4 W/cm2, duty 50%) led to the time-dependent loss of the clotting ability (Figure 2). It has been reported that the γ- irradiation and free-radical oxidation leads to a loss of the clotting ability and increase of the sensitivity to the proteolytic degradation as a result of the fibrinogen aggregate formation [32]. To determine if the US induced aggregation of fibrinogen, the gel-permeation chromatography of fibrinogen was carried out before and after the sonication (Figure 4). The results of gel-permeation chromatography indicated that the formation of fibrinogen aggregates was induced by the US. It has been shown before that aggregation of antithrombin III and streptokinase was also induced by the US [25,26].

Our study demonstrated that the US differentially affected individual proteins and led in case of fibrinogen (Figure 5, line *2*) to increase, and in case of plasminogen (Figure 5, line *3*) and t-PA (Figure 5, line *4*) to decrease of fibrinogenolysis rate. It should be noted that no change in the speed of the fibrinogen hydrolysis was observed using the high-frequency US [16].

It is known that in the presence of fibrinogen, fibrin and their degradation products, the rate of plasminogen activation by t-PA increases due to the formation of tertiary complex: fibrin(ogen) - plasminogen - t-PA [33]. In this case, fibrin potentiates at greater extent an activation rate at the expense of the exposure of the plasminogen and t-PA binding sites that are latent in the fibrinogen molecule. It has been reported that the removal of fibrinopeptides A and B switches interaction of αC-domain from intra- to intermolecular interaction, facilitating the self-association of fibrin [34]. In this regard, the conformational changes occur, which led to the exposure of the plasminogen and t- PA binding sites on the αC-domain surface. At the same time, D-region also contains latent t-PA and the plasminogen binding sites. The exposure of these sites is a result of the noncovalent binding D-D:E [34]. As demonstrated above, US led to the formation of fibrinogen associates with the larger molecular weight. Therefore, it is possible to suggest that the US produces conformational changes in the fibrinogen molecule leading to the exposure of polymerization sites and, as a result, of the plasminogen- and t-PA binding sites. These processes lead to an increase in the rate of the proteolytic degradation of fibrinogen. In turn, a decrease in the fibrinogenolysis rate in cases of prior sonication of plasminogen and t-PA is the result of inactivation of the active forms of these proteins, as it was demonstrated above. The effect of US inactivation enzymes was shown on example of functional model of activation of equimolar mixture of α-chymotrypsinogen and trypsinogen [27]. High sensitivity of t-PA to the US-action is explained by the unique structure of this protein. In comparison with other proenzymes, the single-chain tissue-type plasminogen activator possesses its own catalytic activity. Moreover, the activities of the single- and two-chain t-PA differ only by a factor of 3-9. Existing evidence strongly suggests that Lys^156 ^and Asp^194 ^interact directly in single-chain t-PA and that this interaction stabilizes the active conformation of the enzyme [33].

The obtained results suggest that the preliminary US-treatment of the mixture of fibrinogen with plasminogen leads not to a decrease in the fibrinogenolysis rate, as in case of sonicated plasminogen, but to its increase. The US-treatment of the mixture of fibrinogen with plasminogen, most probably, does not cause any changes in plasminogen, and summary effect is caused by the action on the fibrinogen. It may be assumed that the basic effect of the US on the proteins is caused by the generation of free hydroxyl radicals in the field of US cavitation. Thus, under the condition fibrinogen can act as the "scavenger" of free radicals and, correspondingly, summary effect on the fibrinogen proteolytic degradation for combined US-treatment is determined exclusively by effect on fibrinogen. It is consistent with the fact that fibrinogen appears to be a plasma protein that is most sensitive to *in vitro *metal-ion-catalyzed carbonyl formation [35]. During structural effect induced by therapeutic ultrasound on aqueous solution of six different proteins (cytochrome *c*, lysozyme, myoglobin, bovine serum albumin, trypsinogen and α-chymotrypsinogen A) important role of free radicals, produced by water sonolysis, in the changes of structural order was suggest too [19].

Statistical analysis showed the absence of differences in the kinetic curves of fibrinogenolysis in the following cases of US-treatment: *a*) fibrinogen; *b*) fibrinogen with rt-PA; *c*) fibrinogen with plasminogen and d) fibrinogen, plasminogen and rt-PA (Figure 8). Thus, in case of US-treatment of the protein mixtures effect US on the fibrinogen proteolytic degradation is determined exclusively by effect on fibrinogen. The same results were obtained in the study of the influence of US-processing on the fibrinolysis.

It has been shown before that the US-treatment leads to an increase in the binding t-PA with fibrin [36]. By the scanning electron microscopy showed changes of the fibrin network in the process of US-treatment. Data showed that US leads to formation of many channels in the internal structure of the clot, and irregular disruption and thinning of fibrin fibers at the level of fibrin network structure are observed (Figure 9). It has been demonstrated earlier that disturbances of the fibrin network under the US-action lead to an increase in the penetration of t-PA inside the clot [16]. Due to the increased number of plasminogen/plasmin binding sites, the rate of proteolysis was increased. Obtained data well agree with the layer-by-layer model of the fibrin degradation, in the case of the penetration of the plasminogen activator inside the clot due to the diffusion [37]. Thus, in the control experiment the step-by-step hydrolysis of fibrin was occurred and the large fragments of fibrin were not released into the solution. At US-treatment of fibrin, the proteolysis took place in entire volume of clot and, respectively, the large fragments were released into solution. This is confirmed by the fact that after two hours of hydrolysis in the degradation products of previously US-treated fibrin parallel with D-dimers early fibrin degradation products - fragments X and Y were present, there are not in control. The same differences were observed also at the fibrin clot lysis, induced by circulating and clot-embedded proteases [38].

In summary, under ultrasound treatment of plasminogen and/or t-PA in the presence of fibrin(ogen), the stabilizing effect fibrin(ogen) on given proteins was shown, while an increase in the fibrin(ogen)lysis rate was demonstrated due to both the change in the substrate structure and promoting of the protein-protein complexes formation. This allows us to recommend for the *in vivo *experiments simultaneous introduction of plasminogen activators with the US action and limiting duration of US action by the not more than 5 min.

## Conclusions

Our study demonstrated that the US induces fibrinogen aggregate formation *in vitro*, characterized by loss of clotting ability and greater rate of plasminolysis than native fibrinogen. In case of the US treatment of mixture fibrinogen or fibrin clot with plasminogen, t-PA or both, the rate of proteolytic digestive of fibrin(ogen) increase too, in this case fibrin(ogen) show stabilization effect on plasminogen and t-PA.

## Abbreviations

US: ultrasound; rt-PA: recombinant tissue plasminogen activator; t-PA: tissue plasminogen activator; SDS: sodium dodecyl sulfate; SDS-PAGE: sodium dodecyl sulfate polyacrylamide gel electrophoresis; HIFU: High-intensity focused ultrasound; TCA: trichloroacetic acid; β-ME: β-mercaptoethanol.

## Authors' contributions

EAC performed the majority of the experimental work and drafted manuscript. ISS carried out the gel-permeation chromatography and gel-electrophoresis analysis. IEA participated in the design of the study and helped to draft the manuscript. VMS supervised the experimental work and finalized the manuscript. All authors read and approved the final manuscript.
